# Non-Preemptive Tree Packing

**DOI:** 10.1007/s00453-022-01026-7

**Published:** 2022-08-23

**Authors:** Stefan Lendl, Gerhard Woeginger, Lasse Wulf

**Affiliations:** 1 Department of Operations and Information Systems, University of Graz, Graz, Styria Austria; 2 Department of Computer Science, RWTH Aachen, Aachen, North Rhine-Westphalia Germany; 3 Institute of Discrete Mathematics, Graz University of Technology, Graz, Styria Austria

**Keywords:** Spanning tree packing, Non-preemptive scheduling, Combinatorial optimization, Timevarying graphs, Keeping a network connected over time

## Abstract

An instance of the non-preemptive tree packing problem consists of an undirected graph $G=(V,E)$ together with a weight *w*(*e*) for every edge $e\in E$. The goal is to activate every edge *e* for some time interval of length *w*(*e*), such that the activated edges keep *G* connected for the longest possible overall time. We derive a variety of results on this problem. The problem is strongly NP-hard even on graphs of treewidth 2, and it does not allow a polynomial time approximation scheme (unless P=NP). Furthermore, we discuss the performance of a simple greedy algorithm, and we construct and analyze a number of parameterized and exact algorithms.

## Introduction

**Fig. 1:**
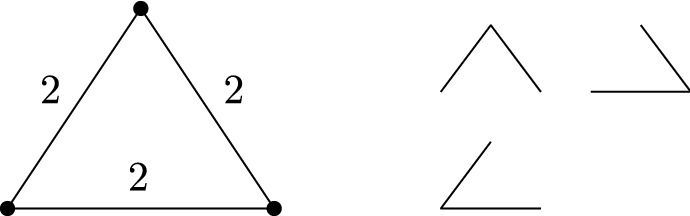
An instance of Nash-Williams’ spanning tree packing problem. The optimal solution packs three spanning trees, as indicated on the right

*The tree packing problem of Nash-Williams.* For a given undirected connected graph $G=(V,E)$ and a weight function $w:E\rightarrow \mathbb {N}$, Nash-Williams [14] considered the optimization problem of packing as many spanning trees as possible into the graph, such that every edge $e \in E$ appears in at most *w*(*e*) of these spanning trees. For example, consider Fig. 1, where *G* is a complete graph on three vertices, and $w(e) = 2$ for all edges. The optimal solution packs three spanning trees. It is not possible to pack more spanning trees, because every edge is already used twice.

Nash-Williams [14] derives a min-max relation for the problem that connects it to certain cut conditions. Building on these results, Cunningham [8] constructs a polynomial time algorithm for the problem by reducing it to a polynomial number of maximum flow problems. Barahona [3] presents another algorithm with a better time complexity.

**Fig. 2:**
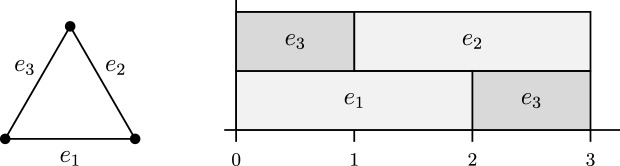
The three edges in the graph on the left hand side have weights $w(e_1)=w(e_2)=w(e_3)=2$. The schedule on the right hand side keeps the graph connected for a total of three time units

The tree packing problem may also be interpreted as a scheduling problem: Every edge $e\in E$ is a resource that can be activated for a total of *w*(*e*) time units. The objective now is to activate the edges in such a way that the graph remains connected for the longest possible overall time. Figure 2 contains a simple illustrating example on the three-vertex cycle. There are three spanning trees (that each consist of two edges), and an optimal schedule uses each of these spanning trees for exactly one time unit. It is easy to see that in every optimal schedule, one of the three edges will be active during the first and the third time slot and will be inactive during the second time slot; in the language of scheduling, we say that the execution of that edge is preempted at time 1 and afterwards resumed at time 2. (In the schedule shown in Fig. 2, edge $e_3$ is the preempted edge.)

*The non-preemptive version of tree packing.* We consider a non-preemptive variant of the above tree packing problem, where the execution of edges must not be preempted: Every edge *e* is activated at some time point $\tau (e)$ chosen by the scheduler, and then remains active without interruption during the full time interval $[\tau (e),\,\tau (e)+w(e)]$. The objective is again to activate the edges in such a way that the graph remains connected for the longest possible overall time. The resulting combinatorial optimization problem is called *non-preemptive tree packing* (N-TreePack for short), and the optimal objective value for a graph $G=(V,E)$ with edge weights $w:E\rightarrow \mathbb {N}_0$ will be denoted ${{\,\mathrm{\mathsf{ntp}}\,}}(G,w)$.

In the example in Fig. 2, every reasonable non-preemptive schedule will activate two of the edges at time 0. As there is no way of keeping the graph connected for more than two time units, the optimal objective value is ${{\,\mathrm{\mathsf{ntp}}\,}}(G,w)=2$.

*Related literature.* The non-preemptive tree packing problem can be understood as a topic with connections to several different areas. First of all, it can be understood as a non-preemptive scheduling problem equipped with a structurally rich global constraint. In scheduling it is the conventional case to assume non-preemption of the scheduled tasks (see the seminal books of Pinedo [18] and Brucker [5]). This shows the importance of handling non-preemption also for more structured scheduling problems like tree packing. In the work of Adjiashvili et al. [1] the computational complexity of time-expanded packing problems with non-preemption constraints is studied and the authors obtain approximation algorithms for several of the studied problem variants.

Our model is also strongly related to the area of *time-varying graphs* (TVG). These are graphs over time, where every edge is equipped with the information at which time it is available. Time-varying graphs are used to model networks, where change in the network topology is an inherent part of the system. This includes for example delay-tolerant networks where information links often fail, like satellite networks, vehicular or passenger networks.

In the seminal paper of Casteigts, Flocchini, Quattrociocchi and Santoro, different classes of TVGs are identified [6]. In particular, class 9 is the class of *constant connected* (also called *1-connected* [15]) TVGs and contains those TVGs which are connected at every point in time. This class was for example used to always guarantee progression of a broadcast operation [16], or to enable consensus in a TVG [13]. Our problem of non-preemptive tree packing can therefore be understood as scheduling the edges in a dynamic network in such a way that the resulting time-varying graph is 1-connected for as long as possible.

Finally, our problem is a modification of the classic tree packing problem (like the classical results of Nash-Williams [14] and Edmonds [10]) with additional global constraints enforcing non-preemptiveness. A similar problem has been studied by Heuvel and Thomassé [12]. Translated to our vocabulary, their work is also an investigation of non-preemptive scheduling, but there are two key differences to our problem: First, they consider a cyclic scheduling where the lifetime of a scheduled element can wrap around from the end to the beginning of the schedule. Second, instead of finding the longest possible time such that the scheduled elements connect the graph at any time point, they consider a dual problem, where the goal is to find the shortest possible time such that the scheduled elements are acyclic at any time point.

*Contributions of this paper.* We analyze the computational complexity and the approximability of non-preemptive tree packing, and we also provide some parameterized and exact algorithms for it. The complexity results are devastating:

- N-TreePack is strongly NP-hard, even on complete bipartite graphs $K_{2,n}$.
- N-TreePack is strongly NP-hard, even on graphs of bandwidth 2.

Since complete bipartite graphs $K_{2,n}$ are series-parallel and since graphs of bandwidth 2 are outerplanar, our results yield strong NP-hardness for essentially all natural subclasses of graphs with treewidth 2. As edges of zero-weight are irrelevant for the objective value of N-TreePack, NP-hardness immediately propagates from graphs to supergraphs; hence our results also imply strong NP-hardness for all the standard families of specially structured graphs, like planar graphs, bipartite graphs, interval graphs, cographs, etc. The only notable exception are the trees and the cactus graphs. Furthermore, we analyze the complexity of cases with small objective values: Deciding whether ${{\,\mathrm{\mathsf{ntp}}\,}}(G,w)\ge \beta$ can be done in polynomial time for $\beta =3$ and is NP-hard for $\beta =7$; the intermediate cases with $\beta \in \{4,5,6\}$ remain open.

With respect to polynomial time approximation, we introduce a simple greedy algorithm which has a worst case guarantee of $n-1$ on *n*-vertex graphs. On cactus graphs it always succeeds in finding an optimal solution, whereas for every non-cactus graph *G* there exist edge weights *w* so that on the input (*G*, *w*) the greedy algorithm fails to find an optimal solution. We show by means of a gap-reduction that (unless P=NP) problem N-TreePack does not allow a polynomial time approximation algorithm with worst case ratio strictly better than 7/6; this of course excludes the existence of a PTAS.

Finally, we derive a number of FPT-results in the area of parameterized complexity. The special case of N-TreePack where both the treewidth and the maximum edge weight are bounded by a constant *k* allows an FPT-algorithm whose running time is linear in |*E*|. (The case where only the treewidth is bounded and the case where only the maximum edge weight is bounded are both para-NP-hard, and hence unlikely to belong to FPT.) Furthermore we design an exact algorithm for N-TreePack whose (exponential) running time is bounded by $|E|!\cdot \text {poly}(|E|)$.

*Organization of the paper.* Section 2 provides central definitions and summarizes the notation. Section 3 contains the NP-hardness results for specially structured graph classes. Sections 4 and 5 contain the negative and positive results for small objective values. Section 6 discusses the greedy algorithm, Sect. 7 states some parameterized and exact algorithms for N-TreePack, and Sect. 8 concludes the paper with some discussion.

## Preliminaries

We write $\mathbb {N}_0= \mathbb {N}\cup \{ 0 \}$ for the set of nonnegative integers. For $a\le b$, the term [*a*, *b*] denotes the *time slot* starting at *a* and ending at *b*. For an integer $t\ge 1$, the time slot $[t-1,t]$ is often called the *t*-th time slot or time slot *t*. Every graph $G=(V,E)$ in this paper is simple, undirected and without loops. We write $V(G) = V$ and $E(G) = E$. For $V'\subseteq V$, the *edge cut* $\delta (V')$ is the set of edges with one endpoint in $V'$ and one endpoint in $V-V'$; for $v\in V$, we write $\delta (v) = \delta (\{ v \})$. We denote by $G[V']$ the *induced subgraph* of *G* by $V'$. By removing a vertex *v* from *G*, we obtain the graph $G-v=G[V-\{ v \}]$. Similarly, for $E'\subseteq E$ and $e\in E$ we have $G-E'=(V,E-E')$ and $G-e=G-\{ e \}$. For all other graph-theoretic concepts used in the paper, we refer the reader to the text book by West [19].

### Formal Problem Definition

An instance for problem N-TreePack is a weighted graph (*G*, *w*), where $G=(V,E)$ and $w:E\rightarrow \mathbb {N}_0$. A *schedule* for instance (*G*, *w*) is a map $\sigma :E\rightarrow \mathbb {N}_0$, that maps each edge *e* to its activation time $\sigma (e)$. For a schedule $\sigma$ and an edge *e*, the *activity interval of e* is $[\sigma (e),\sigma (e)+w(e)]$. For $t\ge 1$, we let

$$\begin{aligned} E^\sigma _t= \{ e\in E:~ \sigma (e)+1 \le t \le \sigma (e)+w(e) \} \end{aligned}$$

denote the set of edges that are active in the *t*-th time slot, and we let $G^\sigma _t=(V,E^\sigma _t)$ denote the graph on vertex set *V* with all the edges that are active in the *t*-th time slot. Finally, we define the *objective value* ${{\,\mathrm{\mathsf{ntp}}\,}}(\sigma )$ of schedule $\sigma$ as the number of time slots $[t-1,t]$ for which $G^\sigma _t$ is connected. When the schedule $\sigma$ is clear from the context, we often simply write $E_t$ and $G_t$ instead of $E^\sigma _t$ and $G^\sigma _t$.

We clarify a few points in this definition: First, the time during which *G* is connected does not necessarily have to be one continuous time interval. Formally, if $C(\sigma ) = \{ t \in \mathbb {N}: G^\sigma _t \text { is connected} \}$ denotes the set of time slots where the graph is connected by schedule $\sigma$, then ${{\,\mathrm{\mathsf{ntp}}\,}}(\sigma ) = |C(\sigma )|$ is simply the cardinality of $C(\sigma )$. Second, there can be a time slot *t*, where $G^\sigma _t$ is connected and contains more edges than a spanning tree, that is, the set of active edges contains a cycle. By our definition, such a time slot also increases ${{\,\mathrm{\mathsf{ntp}}\,}}(\sigma )$ by one. At first glance, it seems like these are important details in the definition of ${{\,\mathrm{\mathsf{ntp}}\,}}(\sigma )$. However, this is not the case – the next lemma shows that one can always assume without loss of generality that an optimal schedule connects the graph for a continuous time interval and does so by using only spanning trees.

### Further Definitions

Throughout the paper, we investigate the (parameterized) complexity of non-preemptive tree packing with respect to different graph classes and structural graph parameters.

The *treewidth* of a graph is a popular parameter in the area of parameterized algorithms and measures in some sense how close the graph is to a tree. It can be defined as the minimum integer $k > 0$ such that *G* is subgraph of a *k*-tree [4]. A *k*-tree is constructed by starting with a $(k +1)$-clique and then repeatedly connecting a new vertex to all vertices of an existing *k*-clique. Two popular subclasses of treewidth-2 graphs are series-parallel graphs and outerplanar graphs. An undirected graph *G* is *series-parallel*, if there exist two vertices *s*, *t* in *G*, such that *G* has a series-parallel decomposition with respect to the source *s* and the sink *t* (see for example [4, p. 22] for details). A graph is *outerplanar* if it has a planar drawing where all the vertices belong to the outer face. Every outerplanar graph is subgraph of a series-parallel graph [4]. The *bandwidth* of a graph is the minimum width of a linear arrangement, where a linear arrangement of a graph is a labeling which assigns to each vertex *v* a distinct integer *f*(*v*) and the width of a linear arrangement is $\max \{ |f(v)-f(u)| : \{ u,v \} \in E \}$. Graphs of bandwidth 2 are outerplanar.

A problem is *strongly NP-hard* if it is NP-hard even if all its numerical parameters are bounded by a polynomial (see Garey and Johnson [11]). For an introduction to parameterized complexity and formal definitions of those concepts we refer the reader to the book of Cygan et al. [9]. A *matroid* is a pair $(E,\mathcal {F})$, where *E* is a finite set called the *ground set* and $\mathcal {F}$ is a family of subsets of *E* with the following properties: (i) We have $\emptyset \in \mathcal {F}$. (ii) For all $F \in \mathcal {F}$ and $F' \subseteq F$ we have $F' \in \mathcal {F}$. (iii) For all $F,F' \in \mathcal {F}$ such that $|F'| < |F|$, there exists an element $e \in F - F'$ such that $F' \cup \{ e \} \in \mathcal {F}$. The sets in $\mathcal {F}$ are called *independent* and a maximal independent set is called a *base*. An important class of matroids are the *graphical matroids*, where *E* is the edge set of some graph and a set $F \subseteq E$ of edges is independent if it does not induce a cycle. The bases in a graphical matroid correspond exactly to the spanning trees of the graph.

### General Insights

#### Lemma 1

For every instance (*G*, *w*) of N-TreePack with ${{\,\mathrm{\mathsf{ntp}}\,}}(G,w)=\beta$, there exists an optimal schedule $\sigma$ which makes the graph connected for the first $\beta$ consecutive time slots (that is $C(\sigma ) = \{ 1, \ldots , \beta \}$) and additionally the graphs $G^\sigma _1,\ldots ,G^\sigma _\beta$ are spanning trees.

#### Proof

Let $G = (V,E)$. We proceed in two steps: First, we prove that one can always make the schedule connected, then we prove that one can always turn the graphs $G^\sigma _1,\ldots ,G^\sigma _\beta$ into spanning trees.

For the first step, assume that $\sigma : E \rightarrow \mathbb {N}_0$ is an optimal schedule with ${{\,\mathrm{\mathsf{ntp}}\,}}(\sigma ) = \beta$, but $\sigma$ is not connected during all of the first $\beta$ time slots, that is $C(\sigma ) \ne \{ 1, \ldots , \beta \}$. Then let *s* be the smallest integer for which $G^\sigma _s$ is disconnected. This corresponds to the *s*-th time slot $[s-1,s]$. By our assumption we have $1 \le s \le \beta$. We now partition the edge set *E* into two sets. The set $E_A$ contains all edges *e* with $\sigma (e) < s-1$ and the set $E_B$ contains all edges *e* with $\sigma (e) \ge s-1$. Now consider the new schedule $\sigma '$ defined by

$$\begin{aligned} \sigma '(e) = {\left\{ \begin{array}{ll} \sigma (e) &{};\text { if } e \in E_A\\ \sigma (e) - 1&{};\text { if } e \in E_B. \end{array}\right. } \end{aligned}$$

In other words, $\sigma '$ is created from $\sigma$ by scheduling all edges of $E_B$ one time unit earlier. Because the edges in $E_A$ connect the graph in $\sigma$ for all time slots $1,\ldots ,s-1$, we immediately see that also all the graphs $G_1^{\sigma '},\ldots ,G^{\sigma '}_{s-1}$ are connected. Furthermore, for every $t \ge s$, we claim that $E^{\sigma '}_t \supseteq E^\sigma _{t+1}$. Indeed, if some edge *e* is contained in $E^\sigma _{t+1}$, then there are two cases: If $e \in E_B$, then it is scheduled one time unit earlier in the schedule $\sigma '$ and hence it is also contained in $E^{\sigma '}_t$. In the other case, if $e \in E_A$, then by definition we have $\sigma (e) < s-1$. But together with the assumption $e \in E^\sigma _{t+1}$, nonpreemptiveness implies that $e \in E^\sigma _t$. Because $\sigma (e) = \sigma '(e)$ we also have $e \in E^{\sigma '}_t$. This proves the claim. In total, we have that if $G^\sigma _{t+1}$ is conneted, then so is $G^{\sigma '}_{t}$ for all $t \ge s$. This proves that ${{\,\mathrm{\mathsf{ntp}}\,}}(\sigma ') \ge {{\,\mathrm{\mathsf{ntp}}\,}}(\sigma )$. If the new schedule $\sigma '$ still does not connect the graph for the first continuous $\beta$ time slots, we can again repeat this procedure, until we arrive at a schedule with the desired property.

For the second step, assume that $\sigma$ is a schedule which already connects the graph for the first $\beta$ time slots (that is $C(\sigma ) = \{ 1, \ldots , \beta \}$), but not all of the graphs $G^\sigma _1,\dots ,G^\sigma _\beta$ are spanning trees. Then let *s* be the smallest integer such that $G^\sigma _s$ contains a cycle. Because $G^\sigma _{s-1}$ does not contain a cycle, there is some edge $e^\star$ on the cycle, which is activated for the first time in the *s*-th time slot $[s-1, s]$, that is $\sigma (e^\star ) = s-1$. Consider the new schedule $\sigma '$ which results from $\sigma$ by scheduling the edge $e^\star$ one time unit later, that is $\sigma '(e^\star ) = \sigma (e^\star ) + 1$ and $\sigma '(e) = \sigma (e)$ for all other edges. Because $e^\star$ was closing a cycle in $G^\sigma _s$, we have that $G^{\sigma '}_s = G^\sigma _s - e^\star$ is still connected. Because the only change in the new schedule $\sigma '$ in comparison to the old schedule $\sigma$ is that we increased the activation time of $e^\star$ by one, we obtain that ${{\,\mathrm{\mathsf{ntp}}\,}}(\sigma ') \ge {{\,\mathrm{\mathsf{ntp}}\,}}(\sigma )$.

In summary, the preceding argument shows that if we select a schedule $\sigma$ with the property that $\sum _{e \in E}\sigma (e)$ is maximal among all the schedules that keep the graph connected for the first consecutive $\beta$ time units, then all the graphs $G^\sigma _1,\dots ,G^\sigma _\beta$ are spanning trees. This proves the lemma. $\square$

**Fig. 3:**
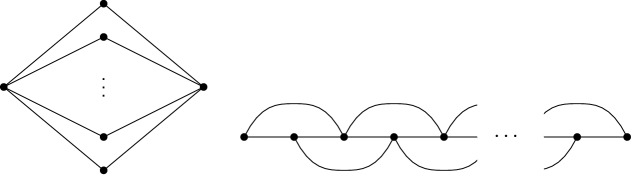
A complete bipartite graph $K_{2,\ell }$ and a graph of bandwidth 2. Problem N-TreePack is NP-hard even on these simple graphs

The ideas presented in Lemma 1 also provide a formal proof that the problem N-TreePack is indeed a nonpreemptive variant of Nash-Williams’ tree packing problem. Indeed, consider the problem of keeping the weighted graph (*G*, *w*) connected, but in the case where edge preemption is allowed. We claim this problem is equivalent to Nash-Williams’ tree packing problem. Indeed, if $(T_1,\dots ,T_\beta )$ is a sequence of $\beta$ spanning trees, then we can schedule in the *i*-th time slot exactly the edges of the *i*-th tree $T_i$ (this is feasible, since preemption is allowed). On the other hand, if we can find a schedule that keeps the graph connected for $\beta$ time units, we can in the same manner as in the proof of Lemma 1 transform the schedule in such a way that all the graphs given by the active edges in the first $\beta$ time slots are spanning trees. Therefore, it is possible to pack $\beta$ spanning trees.

## NP-Hardness

In this section, we establish the NP-hardness of problem N-TreePack for certain families of highly restricted graphs (Fig. 3). All proofs are done by reductions from the strongly NP-hard 3-Partition problem; see Garey & Johnson [11].

Problem 3-Partition:

**Instance:** Positive integers $q_1,\ldots ,q_{3n}$ with sum $\sum _{i=1}^{3n}q_i=nQ$ that satisfy $Q/4<q_i<Q/2$ for all *i*.

**Question**: Is there a partition of these 3*n* numbers into *n* into triplets, such that the numbers in every triplet sum up to *Q*?

In the following, we show that N-TreePack is strongly NP-hard for complete bipartite graphs $K_{2,\ell }$ (Theorem 1). As the graph $K_{2,\ell }$ has unbounded bandwidth, one could hope that the problem becomes easy for graphs of bandwidth 2. But as we show in Theorem 2, also in this case the problem stays strongly NP-hard. As the graph $K_{2,\ell }$ is series-parallel and bandwidth-2 graphs are outerplanar, it follows that problem N-TreePack is intractable even on popular subclasses of treewidth-2 graphs.

### Theorem 1

Problem N-TreePack is strongly NP-hard, even on complete bipartite graphs $K_{2,\ell }$.

### Proof

Let $q_1,\ldots ,q_{3n}$ be an instance of 3-Partition as defined above. Let $\beta = nQ + n - 1$. We construct an instance of N-TreePack on the complete bipartite graph $K_{2,4n-1}$ with bipartition $\{ a,b \}$ and $\{ x_1, \ldots , x_{n-1}, y_1, \ldots , y_{3n} \}$. For $i\in \{1,\ldots ,n-1\}$, we set $w(\{ x_i,a \})= i(Q + 1)$ and $w(\{ x_i,b \})= (n-i)(Q+1)$. For $i\in \{1,\ldots ,3n\}$, we set $w(\{ y_i,a \})= q_i$ and $w(\{ y_i,b \})= \beta$. We claim that the constructed instance of N-TreePack possesses a schedule with objective value $\beta$, if and only if the underlying 3-Partition instance has answer YES.

(Only if) Assume that for the constructed N-TreePack instance there exists a schedule $\sigma$ with objective value $\beta$. Note that for every $i \in \{1,\dots ,n-1\}$, we have $w(\{ x_i,a \}) +w(\{ x_i,b \}) = \beta +1$. Hence the sum of all the edge weights in the graph is $(n-1)(\beta +1) +3n\beta +(\beta -n+1) = 4\beta n$. As every spanning tree for $K_{2, 4n-1}$ has 4*n* edges, each of the connected graphs $G_1,\dots ,G_\beta$ must have exactly 4*n* edges (and must actually be a spanning tree). The fact that the sum of all edge weights is $4\beta n$ furthermore implies that the activity interval of every edge is contained in $[0, \beta ]$. Now consider some vertex $x_i$ with $i\in \{1,\dots ,n-1\}$. Since $w(\{ x_i,a \}) + w(\{ x_i,b \})=\beta +1$, there is exactly one $k\in \{ 1, \ldots , \beta \}$ so that the edge set $E_k$ contains both edges incident to vertex $x_i$. Because there are only two possibilities for the two activity intervals of length $w(\{ x_i,a \}) = i(Q+1)$ and $w(\{ x_i,b \}) = (n-i)(Q+1)$ to cover the whole interval $[0, \beta ]$, we see that either $k=i(Q+1)$ or $k=(n-i)(Q+1)$ holds, and we say that this value *k* is associated with vertex $x_i$. If some value *k* is associated with two distinct vertices $x_i$ and $x_j$, then $G_k$ contains a cycle; a contradiction. We conclude that each of the values $k\in \{ Q+1,2(Q+1),\ldots ,(n-1)(Q+1) \}$ is associated with exactly one of the vertices $x_1,\ldots ,x_{n-1}$. This means that for the corresponding time slots $[k-1,k]$, vertex *a* is connected to vertex *b* via the two edges that are incident to the associated vertex $x_i$. The remaining $\beta -n+1$ time slots form *n* (maximal) intervals each of length *Q*. It is easily verified that during each such interval exactly three vertices $y_i$, $y_j$, $y_{\ell }$ ensure the connection between *a* and *b*, and that the weights of the three edges $\{y_i,a\}$, $\{y_j,a\}$, $\{y_{\ell },a\}$ satisfy $q_i+q_j+q_{\ell }=Q$. Hence the corresponding triplets form a solution for the 3-Partition instance.

(If) Now assume that the 3-Partition instance has a solution. For $1\le i\le n-1$, we activate edge $\{ x_i,a \}$ at time 0 and edge $\{ x_i,b \}$ at time $i(Q+1)-1$. For $1\le i\le n$, we activate edge $\{ y_i,b \}$ at time 0; finally, the edges $\{ y_i,a \}$ are grouped into triplets according to the solution of the 3-Partition instance and scheduled as indicated in the proof of the (only if) part. $\square$

### Theorem 2

Problem N-TreePack is strongly NP-hard, even on graphs of bandwidth 2.

### Proof

Let $q_1,\ldots ,q_{3n}$ be an instance of 3-Partition as defined above. We construct an instance of N-TreePack as follows. The graph *G* has $4n+1$ vertices $u_0,\ldots ,u_n$ and $v_1,\ldots ,v_{3n}$. We will sometimes denote vertex $u_k$ also by the name $v_{k-n}$, for $1\le k\le n+1$; in particular we use $u_n=v_0$ and $u_{n-1}=v_{-1}$. Furthermore we define $\beta =(2n-1)Q$.

- For $k=0,\ldots ,n-1$, the edge $\{u_k, u_{k+1}\}$ receives weight $w(\{u_k, u_{k+1}\}) = 2(n-k-1)Q$.
- For $k=0,\ldots ,n-2$, the edge $\{u_k, u_{k+2}\}$ receives weight $w(\{u_k, u_{k+2}\})=2(k+1)Q$.
- For $k=1,\ldots ,3n$, the edge $\{v_{k-1}, v_k\}$ has weight $w(\{v_{k-1}, v_k\})=q_k$.
- For $k=-1,\ldots ,3n-2$, the edge $\{v_k, v_{k+2}\}$ has weight $w(\{v_k, v_{k+2}\})=\beta$.

In the ordering $u_0,u_1,\ldots ,u_n,v_1,v_2,\ldots ,v_{3n}$, every edge either connects two adjacent vertices or two vertices at distance 2. Hence, the constructed graph $G=(V,E)$ has bandwidth 2.

We will study schedules $\sigma$ of objective value $\beta$. As the sum of all edge weights in *G* equals $\beta (|V|-1)$, each of the graphs $G^\sigma _1, \dots G^\sigma _\beta$ is a spanning tree and the activity interval of every edge is contained in $[0, \beta ]$. We will discuss the behavior of $\sigma$ on the induced subgraph $H_i = G[\{ u_0, \ldots , u_i \}]$ for $1\le i\le n$. Since the only connections between $H_i$ and the rest of the graph are via the two vertices $u_{i-1}$ and $u_i$, during any time slot $[t-1,t]$ with $1\le t\le \beta$, graph $H_i$ will consist of one or two connected components under schedule $\sigma$. If there is a single connected component, we say that $H_i$ is *fully-connected* during the *t*-th time slot. If there are two connected components (one containing vertex $u_{i-1}$, the other one containing vertex $u_i$), we say that $H_i$ is *semi-connected* during the *t*-th time slot. Note that if $H_i$ is semi-connected during the *t*-th time slot, then the edge $\{u_{i-1}, u_{i+1}\}$ must be active during that slot, as there are no other edges that would be able to connect the component containing $u_{i-1}$ to the rest of the graph.

The graph $H_1$ consists of the vertices $u_0$ and $u_1$ and of the edge $\{u_0, u_1\}$ of weight $(2n-2)Q$. Suppose for the sake of contradiction that $H_1$ is neither fully-connected during the first time slot nor during the $\beta$-th time slot. Then the edge $\{u_0, u_2\}$ (of value 2*Q*) must be contained both in $E_1$ and in $E_{\beta }$, which is impossible. By symmetry, we will henceforth assume that under schedule $\sigma$ the graph $G_1$ is fully-connected during the $\beta$-th time slot. This implies that $\{u_0, u_1\}$ is active during $[Q,\beta ]$ and that $\{u_0, u_2\}$ is active during [0, 2*Q*]. For graph $H_i$ (with $1\le i\le n$) one can show by induction that $H_i$ is semi-connected during the time intervals [0, *Q*], [2*Q*, 3*Q*], ..., $[(2i-2)Q,(2i-1)Q]$ and fully-connected at all other moments in $[0,\beta ]$. The induction uses the following facts and observations on the two edges $\{ u_{i-2}, u_i \}$ and $\{ u_{i-1},u_i \}$ that are in $H_i$ but not in $H_{i-1}$:

- Graph $H_i$ is semi-connected during the first time slot: By the inductive hypothesis we have $H_{i-1}$ semi-connected during the first time slot. If $H_i$ would be fully-connected during the first time slot, we would get $\sigma (\{u_{i-2}, u_i\}) = \sigma (\{u_{i-1}, u_i\})=0$. Since all involved edges have weight $w(e)>Q$, this yields a cycle at time $t=Q+1$ as the desired contradiction.
- Since graph $H_{i-1}$ is semi-connected at time 0, the edge $\{u_{i-2},u_i\}$ must be active at time 0 and hence must be active during $[0,(2i-2)Q]$.
- Graph $H_i$ is fully-connected during the $\beta$-th time slot: Otherwise, $H_i$ is fully-connected neither during the first time slot nor during the $\beta$-th time slot. Then the edge $\{u_{i-1},u_{i+1}\}$ would have to be active for $\beta$ time units.
- Since $H_i$ is fully-connected during the $\beta$-th time slot, the edge $\{u_{i-1},u_i\}$ must be active during the $\beta$-th time slot, and hence must be active during the interval $[(2i-1)Q,\beta ]$.

The induction yields for $i=n$ that the induced subgraph $H_n$ is semi-connected during the time intervals [0, *Q*], [2*Q*, 3*Q*], ..., $[(2n-2)Q,(2n-1)Q]$ (that is, all the intervals of length *Q* that start at an even multiple of *Q*) and fully-connected during the time intervals [*Q*, 2*Q*], [3*Q*, 4*Q*], ..., $[(2n-3)Q,(2n-2)Q]$ (that is, all the intervals of length *Q* that start at an odd multiple of *Q*).

Next, consider the subgraph $G'$ that is induced by the $3n+2$ vertices $v_{-1}=u_{n-1}$, $v_0=u_n$ and $v_1,\ldots ,v_{3n}$. As the edges $\{v_k, v_{k+2}\}$ with $k=-1,\ldots ,3n-2$ all have value $\beta$, there is an active path $P_0$ through the vertices with even index during the full interval $[0,\beta ]$ and there is an active path $P_1$ through the vertices with odd index during $[0, \beta ]$. By the above discussion, graph $H_n$ connects these two paths $P_0$ and $P_1$ to each other during the time intervals [*Q*, 2*Q*], [3*Q*, *Q*4], ..., $[(2n-3)Q,(2n-2)Q]$. The only way for connecting $P_0$ and $P_1$ to each other during the remaining time intervals [0, *Q*], [2*Q*, 3*Q*], ..., $[(2n-2)Q,(2n-1)Q]$ is by using the edges $\{v_{k-1},v_k\}$ with $k=1,\ldots ,3n$ of weight $q_k$. As this groups the numbers $q_1,\ldots ,q_{3n}$ into *n* groups with sum *Q*, we get a solution for the instance of 3-Partition.

Vice versa, if the 3-Partition instance has a solution, then we build a schedule $\sigma$ of objective value $\beta$: For $k=0,\ldots ,n-1$, we activate edge $\{ u_k, u_{k+1} \}$ at time $(2k+1)Q$. For $k=0,\ldots ,n-2$, we activate edge $\{ u_k, u_{k+2} \}$ at time 0. For $k=-1,\ldots ,3n-2$, we activate edge $\{ v_k, v_{k+2} \}$ at time 0. Finally, the edges $\{ v_{k-1},v_k \}$ are grouped into triplets and scheduled as described in the other direction of the proof. $\square$

## A Negative Result for Objective Value Seven

In this section, we show that it is NP-hard to decide whether there exists a schedule of objective value at least 7. The reduction is from the following version of the Hamilton cycle problem; see Akiyama, Nishizeki & Saito [2]

Problem Hamilton-3-reg

**Instance:** A bipartite, 3-regular graph $H'$.

**Question:** Does $H'$ possess a Hamilton cycle?

The reduction is done in two steps. The first step transforms an instance $H'$ of Hamilton-3-reg into a new 4-regular graph *H* with the properties described in Lemma 2. The second step then transforms the 4-regular graph *H* from Lemma 2 into a corresponding instance of problem N-TreePack.

### Lemma 2

There is a polynomial time algorithm that takes an instance $H'$ of Hamilton-3-reg as input and outputs a 4-regular bipartite graph *H* together with an edge $\{u,z\}\in E(H)$ (which we call the *special edge*), so that the following holds:

(i): If $H'$ is a YES-instance of Hamilton-3-reg, then the new graph *H* contains a Hamilton cycle that traverses the special edge $\{u,z\}$.
(ii): If $H'$ is a NO-instance of Hamilton-3-reg, then the new graph *H* has no Hamilton path starting in vertex *u*.

### Proof

**Fig. 4:**
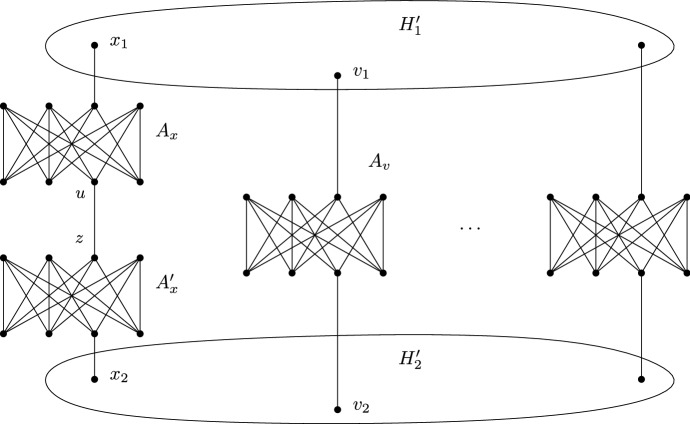
Construction used in the proof of Lemma 2

The reduction is sketched in Fig. 4. Let $H' = (V, E)$. The algorithm chooses some arbitrary vertex $x \in V$. The graph *H* then consists out of the following parts: There are two copies of $H'$, called $H'_1$ and $H'_2$ respectively. For each vertex $v \in V$, we denote by $v_1$ its copy in $H'_1$ and by $v_2$ its copy in $H'_2$. For each vertex $v \in V-\{ x \}$, we take a copy $A_v$ of $K_{4,4}$, remove some edge $\{ u_1, u_2 \}$ from $A_v$, and add edges $\{ v_1,u_1 \}$ and $\{ u_2,v_2 \}$. Finally, we take two copies $A_x$ and $A'_x$ of $K_{4,4}$, remove an edge $\{ u_1,u_2 \}$ from $A_x$ and an edge $\{ u'_1,u'_2 \}$ from $A'_x$, add edges $\{ x_1,u_1 \}$, $\{ u_2,u'_1 \}$, $\{ u'_2,x_2 \}$. The special edge is given by $\{ u, z \}$ with $u := u_2$, and $z := u'_1$. Clearly, *H* is bipartite and 4-regular, so it remains to prove claims (i) and (ii).

For claim (i), assume that $H'$ has a Hamilton cycle $C'$. Then we obtain a Hamilton cycle *C* in *H* using the edge $\{ u,z \}$ in the following fashion: We start at the vertex *u*, and traverse all the vertices of $A_x$ in such an order that we can proceed to go to $x_1$ afterwards. Now we follow the edges of the cycle $C'$, switching alternatingly between $H'_1$ and $H'_2$ after each edge. Precisely, whenever we follow an edge of $C'$ to encounter a new vertex $v_1$ in $H'_1$ ($v_2$ in $H'_2$, respectively), we traverse all the vertices of $A_v$ and go to $v_2$ ($v_1$, respectively) and then follow the next edge of $C'$. Because the graph $H'$ has an even number of vertices (it is bipartite and 3-regular), we will end up at $x_2$. We then complete the tour by traversing all vertices of $A'_x$ and going along the edge $\{ u,z \}$ at the end. This describes a Hamilton cycle of *H* which uses the edge $\{ u,z \}$.

For claim (ii), we prove the contrapositive. Assume that *H* has a Hamilton path $P = (w_1,\dots ,w_n)$ starting at vertex *u*, we have to prove that $H'$ has a Hamilton cycle. Consider the second vertex $w_2$ on the path *P*. Note that if $w_2 = z$, then $w_n$ is in $A_x$. On the other hand, if $w_2$ is in $A_x$, then $w_n$ is in $A'_x$. We first assume that $w_2 \in A_x$ and $w_n \in A'_x$. We claim that the Hamilton path *P* necessarily switches between $H'_1$ and $H'_2$ after every of its edges in $H'_1$ or $H'_2$. Indeed, let $v \ne x$ be a vertex of $H'$ and assume without loss of generality that from the two vertices $v_1$ and $v_2$ the path *P* first encounters $v_1$. If the path does not immediately switch to $v_2$, then it will at a later stage need to visit $v_2$. But in this case, it has to visit $A_v$ after visiting $v_2$, in contradiction to the fact that the path has to return to $A'_x$. This proves that *P* switches between $H'_1$ and $H'_2$ after every egde in $H'_1$ or $H'_2$. Then looking at the edges of *P* which lie in $H'_1$ or $H'_2$, we see that $H'$ has a Hamilton cycle. Finally, an analogous statement holds in the case $w_2 = z$. So we have proven the claim. $\square$

**Fig. 5:**
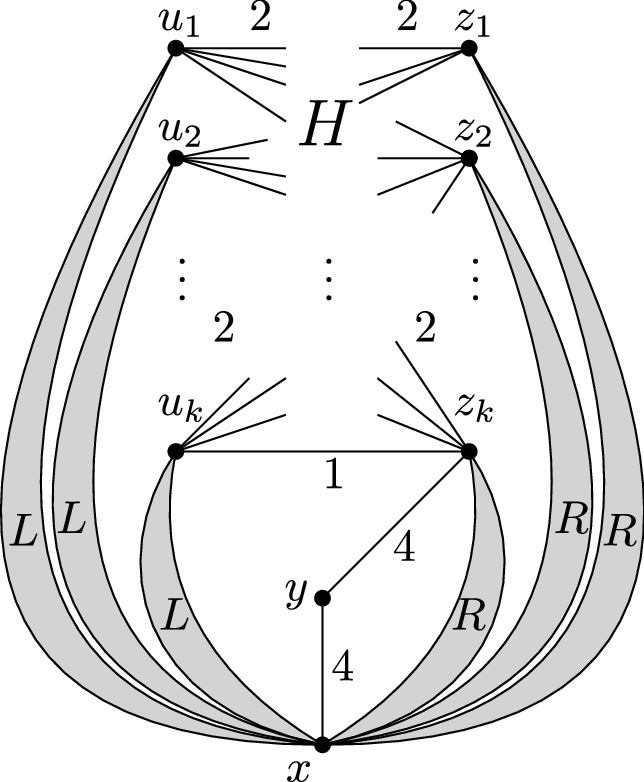
Sketch of the N-TreePack instance (*G*, *w*) obtained from the 4-regular bipartite graph *H*

Now let *H* be a 4-regular bipartite graph as described in Lemma 2. Let $U=\{ u_1, \ldots , u_k \}$ and $Z=\{ z_1, \ldots , z_k \}$ denote the two parts in the bipartition of *H*, and let $\{u_k,z_k\}$ be its special edge. We create an instance (*G*, *w*) of N-TreePack from *H*. An informal sketch of this instance (*G*, *w*) is depicted in Fig. 5. Formally, it is described the following way: The graph *G* has the vertex set

$$\begin{aligned} V(G) = \{ x, y \} \cup U \cup Z \cup \bigcup _{i=1}^k \{ v_{i1}, \ldots , v_{i4} \} \cup \bigcup _{i=1}^k \{ v'_{i1}, v'_{i2} \}. \end{aligned}$$

**Fig. 6:**
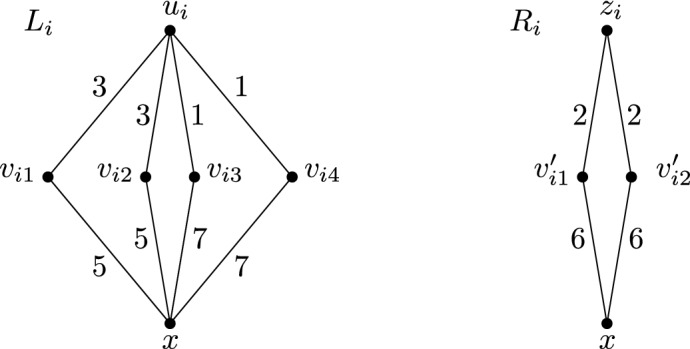
Gadget of type *L* (on the left) and gadget of type *R* (on the right)

Furthermore, the graph *G* has the following edges and edge weights:

- Between the vertex sets *U* and *Z*, the graph *G* has exactly the same edges as the graph *H*. For every such edge *e*, we set $w(e) = 2$, if $e \ne \{ u_k, z_k \}$ and $w(\{ u_k, z_k \}) = 1$.
- For every $i = 1,\dots ,k$, the induced subgraph $L_i = G[\{ x, u_i, v_{i1}, v_{i2}, v_{i3}, v_{i4} \}]$ is called the *i*-*th gadget of type L* and has edges and edge weights as depicted in Fig. 6.
- For every $i = 1,\dots ,k$, the induced subgraph $R_i = G[\{ x, z_i, v'_{i1}, v'_{i2} \}]$ is called the *i*-*th gadget of type R* and has edges and edge weights as depicted in Fig. 6.
- Finally, the two edges $\{ x,y \}$ and $\{ y, z_k \}$ have $w(\{ x,y \}) = w(\{ y, z_k \}) = 4$. The induced subgraph $G[\{ x,y,z_k \}]$ is called the *gadget* *C*.

Now assume that (*G*, *w*) allows some schedule $\sigma$ of objective value 7. During any time slot $[t-1,t]$ with $1\le t\le 7$, the *i*-th gadget of type *L* will consist of either one or two connected components under schedule $\sigma$. If there is a single connected component, we say that $L_i$ is *fully-connected* during the *t*-th time slot. If there are two connected components (one containing *x*, and one containing $u_i$), we say that $L_i$ is *semi-connected* during the *t*-th time slot. In the same manner, during the *t*-th time slot, gadget $R_i$ is either fully-connected or semi-connected with *x* and $z_i$ in different components. Likewise, the gadget *C* is either fully-connected, or semi-connected with *x* and $z_k$ in different components.

### Lemma 3

Let (*G*, *w*) be the instance described above. Every schedule of objective value 7 for (*G*, *w*) satisfies the following.

(i): A gadget of type *R* is fully-connected during time slots 2 and 6, and semi-connected during each of the remaining five time slots.
(ii): For each $i=1,\dots ,k$, there are $t_1,t_2 \in \{ 1,2,4,6,7 \}$ with $t_1\ne t_2$, such that the gadget $L_i$ is fully-connected during time slots $3,5,t_1, t_2$, and semi-connected during each of the remaining three time slots.
(iii): The gadget *C* is fully-connected during time slot 4, and semi-connected during each of the remaining six time slots.

### Proof

Note that the sum of all edge weights in *G* is $8k-1 +32k +16k +8 = 56k+7 = 7(8k + 1) = 7(|V(G)|-1)$. This implies that each of the graphs $G_1,\ldots ,G_7$ is a tree (in particular, it is acyclic) and that the activity interval of each edge is contained in $[0, \beta ]$. For the proof of (i), consider the vertex $v'_{i1}$ in the gadget $R_i$. There is exactly one time slot $[t-1, t]$ during which both edges incident to $v'_{i1}$ are active, and we have $t=2$ or $t=6$. Analogously, there is exactly one time slot $[t'-1, t']$ during which both edges incident to the vertex $v'_{i2}$ are active, and we have $t' = 2$ or $t' = 6$. As $t=t'$ yields the contradiction that $G_t$ has a cycle, we conclude $\{ t,t' \}=\{ 2,6 \}$ and (i) follows. Claims (ii) and (iii) can be proven in the same manner. $\square$

### Lemma 4

Let the graph *H* and the special edge $\{ u,z \}$ be as described in Lemma 2, and let (*G*, *w*) be the corresponding N-TreePack instance. If *H* contains a Hamilton cycle which uses the special edge, then ${{\,\mathrm{\mathsf{ntp}}\,}}(G, w) \ge 7$.

### Proof

Let $e_0 = \{ u, z \} = \{ u_k, z_k \}$ be the special edge. There is a Hamilton cycle *W* using $e_0$. Then the graph $H-E(W)$ is 2-regular, hence there exist pairwise disjoint matchings $M_1, \ldots , M_4$ such that $M_1 \mathbin {\dot{\cup }}M_2 = E(W)$ and $M_3 \mathbin {\dot{\cup }}M_4 = E(H) - E(W)$ and $e_0 \in M_1$. We describe a schedule $\sigma$ with objective value 7:

- All gadgets of type *R* are fully-connected during time slots 2 and 6, and semi-connected otherwise.
- All gadgets of type *L* are fully-connected during time slots 1, 3, 5, and 7, and semi-connected otherwise.
- The gadget *C* is fully-connected during time slot 4, and semi-connected otherwise.
- All edges $e \in M_1 - \{ e_0 \}$ have activity interval [2, 4]. The edge $e_0$ has activity interval [2, 3].
- All edges $e \in M_2$ have activity interval [3, 5].
- All edges $e \in M_3$ have activity interval [0, 2].
- All edges $e \in M_4$ have activity interval [5, 7].

It is easy to see that a schedule $\sigma$ with these properties does indeed exist. Table 1 provides a schematic description of the schedule, by indicating for each number *t*, which edges are scheduled and which gadgets are fully connected during the the *t*-th time slot $[t-1, t]$. Notice that the active edges in *H* form a matching of *H* during the time slots 1,2,3,5,6, and 7, and form a Hamilton path of *H* during the time slot 4. By checking each of the cases $t=1,\dots ,7$, it is easily seen that each of the graphs $G^\sigma _1,\ldots ,G^\sigma _7$ is connected. We therefore conclude that ${{\,\mathrm{\mathsf{ntp}}\,}}(\sigma ) = 7$.

**Table 1:** Description of the schedule $\sigma$ from Lemma 4

time slot						
1	2	3	4	5	6	7
	*R*				*R*	
*L*		*L*		*L*		*L*
			*C*			
		$M_1$	$M_1 - \{ e_0 \}$			
			$M_2$	$M_2$		
$M_3$	$M_3$					
					$M_4$	$M_4$

$$\square $$

### Lemma 5

Let the graph *H* and the special edge $\{ u,z \}$ be as described in Lemma 2, and let (*G*, *w*) be the corresponding N-TreePack instance. If ${{\,\mathrm{\mathsf{ntp}}\,}}(G, w) \ge 7$, then *H* contains a Hamilton path starting at vertex *u*.

### Proof

So assume there exists a schedule $\sigma$ of objective value 7. For a vertex $v \in U \cup Z$, and $t \in \{ 1, \ldots , 7 \}$, let $d_t(v) = |\delta (v) \cap E(H) \cap E_t|$ denote the number of incident edges of *v*, which are both in *E*(*H*) and active during the *t*-th time slot. The strategy of the proof will be to repeatedly deduce some conditions for $d_t(v)$. Let $e_0 = \{ u, z \} = \{ u_k, z_k \}$ be the special edge.

First, recall Lemma 3. Consider vertex $z_i$ for some $i \in \{ 1, \ldots , k \}$. We know that for each $t \in \{ 1, 3, 5, 7 \}$ in the *t*-th time slot both the gadget $R_i$ and the gadget *C* are semi-connected. But of course at least one edge incident to $z_i$ must be active during the *t*-th time slot. Hence $d_1(z_i), d_3(z_i), d_5(z_i), d_7(z_i) \ge 1$. Note that the four edges in $\delta (z_i) \cap E(H)$ each have weight at most 2 (in the case $i \ne k$ we have four times weight 2, and for $i = k$ we have three times weight 2, and $w(e_0) = 1$). For the sake of contradiction, assume $d_1(z_i) > 1$. Then from the four edges in $\delta (z_i) \cap E(H)$ at least two are scheduled at time 0. This is a contradiction to $d_3(z_i), d_5(z_i), d_7(z_i) \ge 1$. Hence $d_1(z_i) = 1$. By the same argument, $d_7(z_i) = 1$. A similar argument shows that $e_0 \not \in E_4$.

Next, consider the graph $G_1$ of active edges in the first time slot. We know that $d_1(z_i) = 1$ for all $i= 1,\dots , k$. Hence the induced subgraph $G_1[U \cup Z]$ on 2*k* vertices is acyclic, has *k* edges, and therefore has *k* connected components. But because $G_1$ is connected, and because all the gadgets of type *R* and the gadget *C* are semi-connected during time slot 1, this implies that every single gadget of type *L* is actually fully-connected during time slot 1. The same argument holds for time slot 7. In total, together with Lemma 3, we have that a gadget of type *L* is fully-connected during the *t*-th time slot, if and only if $t \in \{ 1, 3, 5, 7 \}$. This in turn implies that for all $i = 1,\dots , k$, one has $d_2(u_i), d_4(u_i), d_6(u_i) \ge 1$. The two facts $d_2(u_i) \ge 1$ and $d_6(u_i) \ge 1$ together imply $d_4(u_i) \le 2$. Likewise, for all $i = 1,\dots ,k$, the two facts $d_1(z_i) = 1$ and $d_7(z_i) = 1$ together imply $d_4(z_i) \le 2$.

Finally, we claim $d_4(u_k) = 1$. In fact, $d_4(u_k) = 0$ is impossible, because gadgets of type *L* are semi-connected during time slot 4. For the sake of contradiction, assume $d_4(u_k) > 1$. We know that $d_2(u_k) \ge 1, d_6(u_k) \ge 1$ and $e_0 \not \in E_4$ and $w(e_0) = 1$. So our assumption $d_4(u_k) > 1$ is only possible if $e_0 \in E_2$ or $e_0 \in E_6$. But for the vertex $z_k$, we also know $d_1(z_k), d_3(z_k), d_5(z_k), d_7(z_k) \ge 1$. This is a contradiction to $e_0 \in E_2 \cup E_6$, hence our assumption was wrong and $d_4(u_k) = 1$. In summary, during time slot 4, all gadgets of type *L* and *R* are semi-connected. We also have for all $i=1,\dots ,k$ that $d_4(u_i) \le 2$ and $d_4(z_i) \le 2$. Furthermore, we have $d_4(u_k) = 1$. However, $G_4$ is connected. These facts together imply that the induced subgraph $G_4[U \cup Z]$ is a Hamilton path in *H* starting at $u_k = u$. $\square$

By combining Lemmas 2, 4 and 5 we get the following summarizing theorem.

### Theorem 3

For N-TreePack it is strongly NP-hard to decide whether there exists a schedule of objective value at least 7. $\square$

All edge weights in the above reduction are in the set $\{ 1, \ldots , 7 \}$. A minor modification yields the following corollary.

### Corollary 1

Problem N-TreePack is strongly NP-hard, even if all edge weights are in $\{ 1, \ldots , 6 \}$.

### Proof

**Fig. 7:**
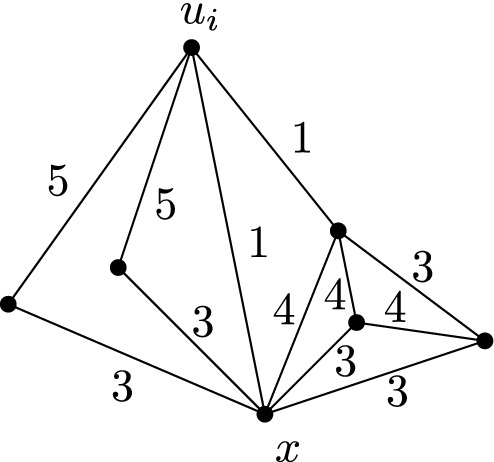
The modified version of the type *L* gadget without edges of weight 7

Edges of weight 7 only show up in the gadget of type *L*. Figure 7 shows a modified version of this gadget that emulates the edges of weight 7 by edges with weights in $\{ 1, \ldots , 6 \}$. One easily verifies that the functionality of the gadget remains the same and that in particular Lemma 3 still holds true. $\square$

As it is NP-hard to distinguish between N-TreePack instances with optimal objective value 6 and N-TreePack instances with optimal objective value 7, we also get the following in approximability result.

### Corollary 2

Unless P=NP, there is no polynomial time approximation algorithm for N-TreePack with worst case guarantee better than 7/6. $\square$

## A Positive Result for Objective Value Three

In Sect. 4, we have established the NP-hardness of deciding whether there exists a schedule of objective value at least 7. As a complementary result, we now show that it can be decided in polynomial time whether there is a schedule of objective value at least 3.

### Theorem 4

For an instance of N-TreePack on a graph with *m* edges, it can be decided in $\mathcal {O}(m^3)$ time whether ${{\,\mathrm{\mathsf{ntp}}\,}}(G,w)\ge 3$.

### Proof

Let $G=(V,E)$. We partition the edge set *E* into set $W_1$ (edges of weight 1), set $W_2$ (edges of weight 2), and set $W_{\ge 3}$ (edges of weight at least 3). In a schedule of objective value 3, we may activate all edges in $W_{\ge 3}$ at time 0. We interpret an edge in $W_2$ as a pair of two edges of weight 1: one of these two edges is scheduled during the middle time slot [1, 2]; the other edge can either be scheduled during slot [0, 1] or during slot [2, 3]. Edges in $W_1$ are scheduled during one of the three slots [0, 1], [1, 2], [2, 3].

By Lemma 1, we may assume that in a feasible schedule of length 3 the graphs $(V,E_t)$ with $t=1,2,3$ are trees. Let $H_3$ be the graph that results from *G* after contracting the edge set $W_{\ge 3}$, and let $H_{23}$ be the graph that results from *G* after contracting the edge set $W_2\cup W_{\ge 3}$. We introduce three matroids:

- The matroid $\mathcal {F}_1$ has the ground set $W_1\cup W_2$. A set $F\subseteq W_1\cup W_2$ is independent in $\mathcal {F}_1$, if and only if *F* is acyclic in graph $H_3$.
- The matroid $\mathcal {F}_2$ has the ground set $W_1$. A set $F\subseteq W_1$ is independent in $\mathcal {F}_2$, if and only if *F* is acyclic in graph $H_{23}$.
- The matroid $\mathcal {F}_3$ has ground set $W_1\cup W_2$, and coincides with $\mathcal {F}_1$.

Recall that a base of a matroid is a maximal independent set. By construction, the bases of $\mathcal {F}_1, \mathcal {F}_2$ and $\mathcal {F}_3$ have the following properties. (A small technicality we have to consider is that $W_2$ and $W_{\ge 3}$ may contain cycles.)

- If a set $F \subseteq W_1 \cup W_2$ is independent in the matroid $\mathcal {F}_1$, then *F* is a base of $\mathcal {F}_1$ if and only if *F* induces a spanning tree of $H_3$ if and only if $F \cup W_{\ge 3}$ contains a spanning tree of *G*.
- If a set $F \subseteq W_1$ is independent in the matroid $\mathcal {F}_2$, then *F* is a base of $\mathcal {F}_2$ if and only if *F* induces a spanning tree of $H_{23}$ if and only if $F \cup W_2 \cup W_{\ge 3}$ contains a spanning tree of *G*.
- If a set $F \subseteq W_1 \cup W_2$ is independent in the matroid $\mathcal {F}_3$, then *F* is a base of $\mathcal {F}_3$ if and only if *F* induces a spanning tree of $H_3$ if and only if $F \cup W_{\ge 3}$ contains a spanning tree of *G*.

Now observe that scheduling an edge of weight 1 is equivalent to choosing in which of the three time slots 1,2 or 3 it is scheduled. Scheduling an edge 2 is equivalent to always schedule it in time slot 2 and choose whether it is scheduled in time slot 1 or 3. From these facts together with Lemma 1, we can deduce that we can connect the graph during time slots 1,2 and 3 (that is ${{\,\mathrm{\mathsf{ntp}}\,}}(G,w)\ge 3$ holds), if and only if there exist three pairwise disjoint subsets $S_1,S_2,S_3$ of $W_1 \cup W_2$, such that $S_t$ forms a base of the matroid $\mathcal {F}_t$ for $t=1,2,3$. This can be checked in $\mathcal {O}(m^3)$ time by using Edmond’s matroid partitioning algorithm [10]. $\square$

By a similar (but simpler) argument we can also decide in polynomial time whether ${{\,\mathrm{\mathsf{ntp}}\,}}(G,w)\ge 2$. Deciding whether ${{\,\mathrm{\mathsf{ntp}}\,}}(G,w)\ge 1$ is trivial. The complexity of deciding whether ${{\,\mathrm{\mathsf{ntp}}\,}}(G,w)\ge \beta$ remains open for $\beta \in \{4,5,6\}$.

## The Greedy Algorithm

We introduce a greedy algorithm that maintains connectivity by always activating edges of the largest possible weight. Formally, we let $F_t\subseteq E_t$ denote the set of edges whose activity intervals end at time *t*. By $U_t=E-(E_1\cup E_2\cup \cdots \cup E_t)$ we denote the set of edges that have not been used and activated before time *t*.

Now the Greedy algorithm starts its work by initializing $E_0:=\emptyset$, $F_0:=\emptyset$, and $U_0:=E$. For $t\ge 0$, the set $E_{t+1}$ for time slot $[t,t+1]$ is computed as follows. If the graph $(V,E_t-F_t)$ is a tree, we set $E_{t+1}:=E_t$. If the graph $(V,E_t-F_t)$ is a forest with *c* components, we turn it into a tree by adding a maximum weight subset $A\subseteq U_t$ of cardinality $c-1$; then we set $E_{t+1}:=(E_t-F_t)\cup A$. In case no such set *A* exists, the Greedy algorithm terminates. (The set *A* can be computed for instance by applying Kruskal’s algorithm for maximum spanning trees; ties are broken arbitrarily.)

### Theorem 5

For every graph $G=(V,E)$ on *n* vertices and for every $w:E\rightarrow \mathbb {N}_0$, the Greedy algorithm computes a schedule of length at least ${{\,\mathrm{\mathsf{ntp}}\,}}(G,w)/(n-1)$. Furthermore, there exist instances on which the schedule computed by the Greedy algorithm is a factor $\lfloor n/2\rfloor$ below the optimal objective value.

### Proof

For the positive result, we consider the time slot $[T,T+1]$ at which Greedy terminates. Then the graph $(V,(E_T-F_T)\cup U_T)$ is not connected. We consider the vertex set $C\subseteq V$ of one of the components of that graph, and the corresponding edge cut $\delta (C)$. Then the weight $w(\delta (C))=\sum _{e\in \delta (C)}w(e)$ yields a trivial upper bound for the optimal objective value:

1 $$\begin{aligned} {{\,\mathrm{\mathsf{ntp}}\,}}(G,w) ~\le ~ w(\delta (C)) \end{aligned}$$

Since every edge set $E_j$ with $1\le j\le T$ induces a tree, we have $|E_j|=n-1$ and hence $|E_j\cap \delta (C)|\le n-1$. As all edges in the cut $\delta (C)$ have been activated and run to completion before the time slot $[T,T+1]$, we conclude $T\ge w(\delta (C))/(n-1)$, which together with (1) yields the desired approximation guarantee.

For the negative result, we consider the complete graph $K_n=(V,E)$ on *n* vertices with weights $w(e)=1$ for all $e\in E$. A folklore result (see for instance Palmer [17]) says that the maximum number of edge-disjoint spanning trees that can be packed into $K_n$ is $\lfloor n/2\rfloor$. This implies ${{\,\mathrm{\mathsf{ntp}}\,}}(K_n,w)=\lfloor n/2\rfloor$. On the other hand, if the Greedy algorithm at time 0 activates the $n-1$ edges in the edge cut $\delta (v)$ for some $v\in V$, the objective value of the resulting schedule equals 1. $\square$

### Theorem 6

For every connected graph $G=(V,E)$, the following two statements are equivalent.

(i): *G* is a cactus graph.
(ii): For every choice $w:E\rightarrow \mathbb {N}_0$ of edge weights, the Greedy algorithm solves the N-TreePack instance (*G*, *w*) to optimality.

**Fig. 8:**
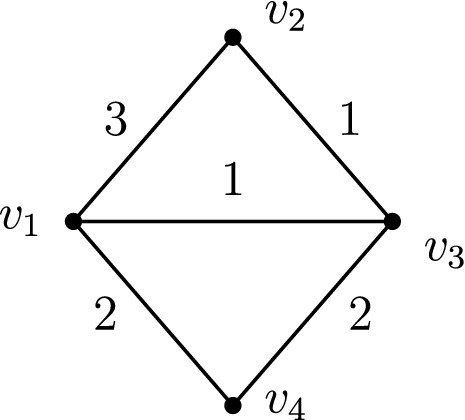
An instance $(H,w_H)$ on which the Greedy algorithm fails

### Proof

We first show that (i) implies (ii). Since cut-vertices split an N-TreePack instance into smaller instances that do not interact with each other, it is sufficient to prove the statement for two-connected cactus graphs. Hence we will assume that *G* is a cycle on *n* vertices, and we let $e_1,\ldots ,e_n$ denote the edges in the cycle ordered by increasing weight so that $w(e_1)\le w(e_2)\le \cdots \le w(e_n)$. It is easily seen that the optimal objective value for (*G*, *w*) equals $\min \{w(e_1)+w(e_2),\,w(e_3)\}$. As the Greedy algorithm activates the $n-1$ edges $e_2,\ldots ,e_n$ at time 0 and activates the final edge $e_1$ at time $w(e_2)$, it yields the optimal objective value.

In order to show that (ii) implies (i), we first consider the graph *H* on the four vertices $v_1,v_2,v_3,v_4$ and the edge weights $w_H$ depicted in Fig. 8. As the Greedy algorithm at time 0 activates the three edges $\{v_1,v_2\}$, $\{v_1,v_4\}$, $\{v_3,v_4\}$ (that carry the weights 3, 2, 2, respectively), at time 2 there remains no further possibility of connecting vertex $v_4$ to the rest of the graph; hence Greedy generates a schedule of value 2. On the other hand, the optimal schedule results by activating the three edges $\{v_1,v_2\}$, $\{v_1,v_3\}$, $\{v_3,v_4\}$ at time 0, by activating edge $\{v_1,v_4\}$ at time 1, and by activating edge $\{v_2,v_3\}$ at time 2; the corresponding optimal objective value is ${{\,\mathrm{\mathsf{ntp}}\,}}(H,w_H)=3$. Hence Greedy fails to solve this instance $(H,w_H)$ to optimality.

Now let $G=(V,E)$ be an arbitrary connected graph that is not a cactus. This implies that *G* does contain a subdivision $(V',E')$ of the four-vertex graph $H=(V_H,E_H)$ in Fig. 8. Let $E''\subseteq E$ be a subset of cardinality $|V-V'|$, so that the graph $(V,E'\cup E'')$ is connected. We construct edge weights $w:E\rightarrow \mathbb {N}_0$ as follows. For every edge $e\notin E'\cup E''$ we set $w(e)=0$, and for every edge $e\in E''$ we set $w(e)=3$. Finally, we fix the weights *w*(*e*) of the edges $e\in E'$ so that they emulate the weights $w_H:E_H\rightarrow \mathbb {N}_0$ in the four-vertex graph *H*; if an edge $e\in E'$ belongs to the subdivision of some edge $f\in E_H$, we define $w(e)=w_H(f)$. It is easily verified that the resulting instance (*G*, *w*) satisfies ${{\,\mathrm{\mathsf{ntp}}\,}}(G,w)=3$, whereas the Greedy algorithm only yields an objective value of 2. $\square$

## Parameterized Complexity

In this section, we show that problem N-TreePack is fixed parameter tractable with respect to various parameters. As the problem is already NP-hard even for highly restricted graphs, we can only hope for positive parameterized complexity results for quite restricitive graph parameters, or when additionally the edge weights are restricted. Note that Theorems 1 and 3 imply the NP-hardness of problem N-TreePack, even if either the treewidth or the edge weights are bounded by a constant.

### Theorem 7

Problem N-TreePack is fixed parameter tractable with respect to $w_{\max } + t$, where $w_{\max }$ is the maximum edge weight and *t* the treewidth of the input graph.

### Proof

As the graph $G=(V,E)$ has treewidth *t*, there is a vertex $v\in V$ of degree at most *t* (here we use the property that *G* is a simple graph, that is, it has no parallel edges.). As every edge incident to *v* has weight at most $w_{\max }$, we conclude ${{\,\mathrm{\mathsf{ntp}}\,}}(G,w)\le w_{\max }^2$. For every $T=1,\ldots ,w_{\max }^2$, we construct a formula $\Phi _T$ in monadic second-order graph logic $\text {MSO}_2$, so that $\Phi _T$ is satisfiable if and only if there exists a schedule of objective value at least *T*. We introduce Boolean variables $x_{e,t}$ for every $e\in E$ and every $t\in \{1,\dots ,T\}$ to denote whether $e\in E_t$. The following statement can be formulated in $\text {MSO}_2$ by routine methods (see for example [9, chapter 7.4]):

$$\begin{aligned} \exists \sigma : E\rightarrow \{0,\ldots ,&T\} ~~\forall e\in E ~~\forall t\in \{1,\ldots ,T\}: \\&x_{e,t} \iff \left( \sigma (e) < t \le \sigma (e) + w(e)\right) \\&\wedge \forall t\in \{1,\ldots ,T\}: \{ e\in E \mid x_{e,t} \} \text { is a spanning tree.} \end{aligned}$$

Now Courcelle’s theorem [7] implies that the satisfiability of $\Phi _T$ can be checked in linear time for every $T=1,\ldots ,w_{\max }^2$. $\square$

### Theorem 8

On input graphs $G=(V,E)$, problem N-TreePack is solvable in exponential time $\mathcal {O}(|E|^2\cdot |E|!)$.

### Proof

By Lemma 1, we may assume that in an optimal schedule $\sigma$ of length *T* all graphs $G_1^\sigma , \dots , G_T^\sigma$ are trees. As these trees are uniquely determined by the ordering in which $\sigma$ activates the edges, we only need to check and evaluate |*E*|! cases. Each case is easily checked and evaluated in $\mathcal {O}(|E|^2)$ time. $\square$

### Theorem 9

Problem N-TreePack is fixed parameter tractable with respect to the size *k* of a feedback edge set. There is a kernel with $\mathcal {O}(k)$ vertices and edges.

### Proof

Note that the input graph $G=(V,E)$ satisfies $|E|\le |V|-1+k$. We first prove an auxiliary observation on the largest edge weight $w_{\max }$ in the graph: We claim that $|V|\ge k+2$ implies ${{\,\mathrm{\mathsf{ntp}}\,}}(G,w)\le w_{\max }$. Consider an optimal schedule as in Lemma 1, so that $|E_1|=|V|-1$. As every edge in $E_1$ is certainly inactive from time $w_{\max }$ onwards, there remain at most $|E|-|E_1|=|E|-|V|+1\le k$ edges that could become active during the next time slot $[w_{\max },w_{\max }+1]$. As a spanning tree needs at least $|V|-1\ge k+1$ edges, this proves the auxiliary observation. As a consequence we get that in an optimal schedule an edge with weight $w_{\max }$ without loss of generality may be activated at time 0.

Now the kernelization procedure is clear: As long as $|V|\ge k+2$ holds, we contract an edge with largest edge weight. Note that the contraction maintains the inequality $|E|\le |V|-1+k$. The resulting kernel satisfies $|V|\le k+1$ and $|E|\le 2k$. $\square$

The last theorem of this section shows that problem N-TreePack is tractable on instances that in a certain sense are close to the preemptive tree packing problem of Nash-Williams.

### Theorem 10

Let (*G*, *w*) be an instance of N-TreePack on *m* edges, so that $m-k$ edges have weight 1 and the remaining *k* edges have weight at most *k*. Then an optimal solution can be found in $\mathcal {O}(k^{2k}m^3)$ time.

### Proof

Let $E'=\{ e\in E: w(e)\ne 1 \}$. For a schedule $\sigma$ of objective value *T*, we denote by $D_\sigma = \{ t: E_t^\sigma \cap E'\ne \emptyset \}$ the set of time slots $[t-1,t]$ during which at least one edge of $E'$ is active. For every $t\le T$ with $t\notin D_\sigma$, graph $G_t=(V,E_t)$ is a connected graph in which all edges have weight 1. For each such *t* with $t \not \in D_\sigma$, we introduce another schedule $\pi$, where the edge set $E_t$ is scheduled in the last time slot $[T-1, T]$ instead of the *t*-th time slot, and all edges activated during [*t*, *T*] are activated one time unit earlier instead. Formally, schedule $\pi$ is such that

$$\begin{aligned} (G^\pi _1,\dots , G^\pi _T) = (G^\sigma _1, \dots G^\sigma _{t-1}, G^\sigma _{t+1}, \dots , G^\sigma _T, G^\sigma _t). \end{aligned}$$

Observe that $\pi$ is also a schedule of objective value *T*. By repeating this procedure often enough, we conclude that there always exists an optimal schedule $\sigma$ so that $D_\sigma \subseteq \{ 1, \ldots , k^2 \}$. Due to Lemma 1, we can additionally require that each of $G_1, \dots , G_T$ is acyclic.

Therefore, the following is an algorithm to solve problem N-TreePack: Iterate over all possible $k^{2k}$ choices of $(\sigma (e))_{e \in E'} \in \{ 1, \ldots , k^2 \}^k$. For each fixed choice, the only edges left to schedule are edges of weight one. This can be done optimally the following way: For $t = 1,\dots , k^2$, let $W_t = \{ e \in E' : \sigma (e) < t \le \sigma (e) + w(e) \}$ be the set of edges in $E'$, which are active during the *t*-th time slot. If some $W_t$ has a cycle, we immediately skip to the next choice of $(\sigma (e))_{e \in E'}$. Otherwise, consider the matroid $\mathcal {F}_t = \{ F \subseteq E(G)-E' : W_t \cup F \text { is acyclic} \}$. We extend the definition of $\mathcal {F}_t$ to $t > k^2$ by setting $W_t = \emptyset$ in this case. The matroid $\mathcal {F}_t$ is isomorphic to the graphic matroid of *G* after contracting each connected component of $W_t$ to a single vertex. Now we run Edmond’s Matroid Partitioning algorithm [10] to determine the maximal $T'\in \mathbb {N}_0$ such that $E(G)-E'$ contains disjoint sets $F_1, \dots , F_{T'}$ such that $F_t$ is a base of $\mathcal {F}_t$ for all $t \in \{ 1, \ldots , T' \}$ (that is, we solve the problem of packing as many bases as possible into a matroid). As Edmond’s Matroid Partitioning algorithm runs in $\mathcal {O}(m^3)$ time, the claimed time complexity follows. $\square$

## Conclusion

We have analyzed the computational complexity and the algorithmic behavior of non-preemptive tree packing. The problem is strongly NP-hard even on highly structured and extremely simple graph classes, and we only have a handful of positive results.

There remain many open questions.  (Q1) We have shown that N-TreePack can be approximated in polynomial time within a factor of $n-1$, and that no approximation factor better than 7/6 is possible (unless P=NP). Where is the true approximation threshold? In particular, we would like to know whether our problem allows a polynomial time approximation algorithm with some constant worst case guarantee. A major step towards an answer might be the analysis of the gap between the non-preemptive optimum and the polynomially computable preemptive optimum.  (Q2) If all edge weights are equal to 1, problem N-TreePack coincides with the preemptive problem version and hence is polynomially solvable. On the other hand the problem is NP-hard, if all edge weights are in $\{ 1, \ldots , 6 \}$. What is the complexity of N-TreePack, if all edge weights are in $\{1,2\}$?  (Q3) The problem of deciding whether ${{\,\mathrm{\mathsf{ntp}}\,}}(G,w)\ge \beta$ is polynomially solvable for every $\beta \le 3$ and NP-hard for every $\beta \ge 7$. What is the complexity of this question for $\beta \in \{4,5,6\}$?
